# Sex and Gender Differences in Central Nervous System-Related Disorders

**DOI:** 10.1155/2016/2827090

**Published:** 2016-05-30

**Authors:** Emanuela Zagni, Lucia Simoni, Delia Colombo

**Affiliations:** ^1^Novartis Farma S.p.A., Largo Umberto Boccioni 1, 21040 Origgio, Italy; ^2^MediNeos Observational Research, Viale Virgilio 54/U, 41123 Modena, Italy

## Abstract

There are important sex differences in the brain that seem to arise from biology as well as psychosocial influences. Sex differences in several aspects of human behavior and cognition have been reported. Gonadal sex steroids or genes found on sex chromosomes influence sex differences in neuroanatomy, neurochemistry and neuronal structure, and connectivity. There has been some resistance to accept that sex differences in the human brain exist and have biological relevance; however, a few years ago, it has been recommended by the USA National Institute of Mental Health to incorporate sex as a variable in experimental and clinical neurological and psychiatric studies. We here review the clinical literature on sex differences in pain and neurological and psychiatric diseases, with the aim to further stimulate interest in sexual dimorphisms in the brain and brain diseases, possibly encouraging more research in the field of the implications of sex differences for treating these conditions.

## 1. Introduction

In 2001, the Institute of Medicine, a branch of the National Academy of Sciences in the USA, stated that many aspects of brain functioning exhibit important, although yet poorly understood, sex differences [1]. Ten years later, a workshop on “Sex Differences in Brain, Behavior, Mental Health, and Mental Disorders” organized by the National Institute of Mental Health concluded that there are striking sex differences in the brain and that it is necessary to incorporate sex as a variable in experimental and clinical studies [2]. Since then, several other governments' funding agencies in the United States and Europe have recommended investigating the impact of sex and/or gender in order to increase the understanding of normal brain development and functioning, as well as CNS-related diseases.

Sex differences in several aspects of human behavior and cognition have been reported, but it is not clear whether these differences arise from biology or societal influences. However, it has been shown that there are sex differences in the brain determining the expression of male- or female-typical behaviors [3, 4]. Gonadal sex steroids or genes found on sex chromosomes influence sex differences in neuroanatomy, neurochemistry and neuronal structure, and connectivity. There has been some resistance to accept that sex differences in the human brain exist and have biological relevance [5]; however, sex differences in the brain are more pervasive than it may be thought.

Exploring sexual dimorphisms in the brain is important for their impact and therapeutic implications for many neurological and psychiatric diseases. An important aspect of the gender bias in the relative risk of mental illness is the preponderance of developmental onset disorders, such as Autism Spectrum Disorders (ASDs) and Attention Deficit and Hyperactivity Disorders (ADHD) in males, whereas adult onset disorders have a higher frequency in females [3, 6, 7]. Important sex differences have been reported in trauma-related disorders and major depression [8], in anxiety and depressive disorders [9], in autoimmune diseases affecting the nervous system [10], and in neurodegenerative disorders [11]. Furthermore, there is extensive literature on sex differences in cognitive decline and Alzheimer disease, recently reviewed by Li and Singh [12]. Last but not the least, in recent years, there has been a substantial increase in research regarding sex differences in pain, as recently reviewed by Bartley and Fillingim [13].

Despite all these profound sex differences, males remain the research subject of choice in neuroscience [3]. In this paper, we explore sex differences in neurological and psychiatric disorders by reviewing clinical data. Reviewing such data may highlight the value of studying sex differences in order to better understand the underlying mechanisms and hopefully encouraging further sex-specific clinical research eventually aimed at developing more targeted therapy, especially for those diseases where sex differences are most prominent.

## 2. Neurodevelopmental Disorders

ASD may be considered the prototypical sex-biased neurodevelopmental disorder, with a sex ratio of four males for every female that reaches eleven to one in high-functioning autism, though the studies of the last two decades show a trend of decreasing male predominance [14–16].

Schaafsma and Pfaff [7] have recently explored the potential genetic, hormonal, and environmental mechanisms underlying ASD's male prevalence. ASD is now recognized as a genetic disorder, but most of the genes implicated in the disorder are not located on sex chromosomes. However, it remains possible that genes on the Y chromosome interact with ASD susceptibility genes to contribute to the autistic phenotype. Likewise, genes located on the X chromosome that escape X-inactivation or are susceptible to skewed or mosaic inactivation, or imprinting may contribute to the male sex bias. All these mechanisms probably contribute to etiology, rather than being simply causative to ASD, since environments, both internal and external, also play important roles in ASD's etiology. Developmental exposure to steroid sex hormones has also been postulated as a contributor to ASD phenotypes in males, but the data are inconsistent and suggest that increased steroid exposure alone is unlikely to be responsible for the male predominance in ASD. The same authors have also explored the possibility of an interaction between sex steroids, immune factors or prenatal stressors, and susceptibility genes in predisposing males to develop ASD, an explanation that may be valid for other psychiatric and neurological disorders characterized by a male sex bias, including ADHD.

Actually, many of the same considerations put forth by Schaafsma and Pfaff for autism are addressed by Davies [6] in his recent review of the biological mechanisms that may, and in some cases have been shown to, contribute to the sex bias in ADHD, which affects ten times more males than females.

It has been hypothesized that the fact that ASDs are diagnosed more frequently in boys than in girls may be due to an underidentification of females with autism due to “females' camouflaging.” A very recent Polish study found that high-functioning females with autism present better on nonverbal (gestures) mode of communication than boys with autism [17]. This may be because they are effective at camouflaging other diagnostic features. This may pose a risk of underdiagnosis or not receiving the appropriate diagnosis for this population. Since girls with autism presented a higher Gesture Index than boys with autism, which was interpreted as gestures with increased energy, more “vivid” and thus probably more noticeable by an examiner (a human), the authors hypothesize that such nonverbal communication may be noted as not autistic.

Only a modest body of research exists examining sex differences in ASD characteristics. An underrepresentation of females in the ASD literature may have led to limited knowledge of differences in social function across the sexes. Reviewing the recent literature, gender differences in the phenotype of ASD are controversial. According to the “extreme male brain” (EMB) theory, there are typical male and female cognitive profiles (“brain types”) in the general population, in two domains: empathizing (the drive and ability to identify a person's thoughts and feelings and to respond to these with an appropriate emotion) [18] and systemizing (the drive and ability to analyze or build systems) [19]. Typical females, on average, exhibit more empathizing and less systemizing compared to typical males, and people with autism show an extreme of this “male profile” [20, 21]. A study by Baron-Cohen et al. provides strong support for the EMB theory of autism [22], showing that the cognitive profiles of both males and females with autism are shifted towards and beyond the typical male distribution, and normative sex differences in these profiles are attenuated in autism. A large study on almost 2500 children with autism, aimed at examining differences in behavioral symptoms and cognitive functioning between males and females with ASD, showed that females had greater social communication impairment, lower levels of restricted interests, lower cognitive ability, weaker adaptive skills, and greater externalizing problems relative to males. IQ reductions mediated greater social impairment and reduced adaptive behavior in females but did not mediate reductions in restricted interests or increases in irritability [23]. In another study on approximately 350 ASD subjects among males and females, total difficulties scored significantly higher in girls with ASD than in boys (at the Strength and Difficulties Questionnaire) [24]. An interesting, though preliminary, study of gender differences in autobiographical memory in children with ASD suggested that a deficit in specific memory retrieval was more characteristic of male participants, while females generated more detailed and emotional memories than males. Females also demonstrated superior verbal fluency scores [25]. Males with autism seem to exhibit more repetitive behaviors than females with autism [26]. On the other hand, other studies did not reveal significant gender differences in autism symptoms, developmental quotient, children's adaptive skills, and behavior problems and suggest a similar phenotype in males and females [27, 28]. Further research is needed to examine sex differences across development.

Also brain morphology as yet remains unclear and requires future dedicated investigations. A recent study [6] provides evidence of structural brain gender differences in young children with ASD that may contribute to the difference in phenotypic disease manifestations between males and females observed in some cohorts.

## 3. Affective Disorders

Sex differences are prominent in mood and anxiety disorders. Women are roughly twice as likely to suffer from anxiety disorders, such as panic disorder and trauma-related disorders (e.g., posttraumatic stress disorder) and are more likely to suffer from major depression than men [29, 30]. Several biological processes are thought to be involved in the predisposition of women to depression, including genetically determined vulnerability, hormonal fluctuations related to various aspects of reproductive function, and a high sensitivity to such hormonal fluctuations in brain systems that mediate depressive states. Furthermore, psychosocial events such as role-stress, victimization, sex-specific socialization, internalization coping style, and disadvantaged social status have all been considered to be contributors to the increased vulnerability of women to depression.

Actually, extensive research has consistently found that women are more likely to be diagnosed with depression and demonstrate more distress in the form of depressive symptoms than men [29–34]. Women are more susceptible than men to stress-induced depression and to changes in photoperiod (more than 80% of individuals with seasonal affective disorder are women). Depression in women may develop during different phases of the reproductive cycle (premenstrual dysphoric disorder, depression during pregnancy, postpartum depressive conditions, and menopausal depression) and reproductive events such as infertility, miscarriage, oral contraceptives, and hormone replacement treatment have also been reported to cause depression in women [35]. How sex differences at the molecular and cellular level can contribute to sex differences in disease vulnerability and severity has been recently discussed [8]. Altemus et al. [9] have reviewed sex differences in anxiety and depressive disorders from a clinical perspective underlining the role of developmental stages. They propose that sex differences that promote reproductive success also result in differential risk for psychopathology.

It has been shown that affective disorders are commonly associated with a dysregulation of the hypothalamus-pituitary-adrenal (HPA) axis [35]. Actually, sex differences in the incidence of major depressive disorders correlate with sex differences in HPA axis function. Organizational and activational effects of gonadal steroid hormones have been shown on the regulation of HPA axis function and may underlie the increased risk of affective disorders in women. Further, prenatal stress and prenatal overexposure to glucocorticoids can impact adult behaviors and neuroendocrine responses to stress. Consistently, the clinical benefits of antidepressants are also associated with the normalization of the dysregulated HPA axis, and genetic polymorphisms have been found in some genes involved in controlling the stress response. Thus, the authors suggest that the impact of gender must be taken into consideration when considering any therapeutic approach for affective disorders.

Kessler [29] underlined the fact that if it is true that gender differences first emerge in puberty, it has also been shown that other experiences related to changes in sex hormones (pregnancy, menopause, use of oral contraceptives, and use of hormone replacement therapy) do not significantly influence major depression. These observations suggested that the key to understand the higher rates of major depression among women than men may lie in the joint effects of biological vulnerabilities and environmental provoking experiences. It has also been postulated that gender, rather than sex, contributes to the manifestation of stress as depression [36–38]. Gender is a socially constructed status that is not only distinct from biological sex, but may be more influential in determining mental well-being than sex [39]. The social construction of gender and sex is interconnected and conditioned by membership in multiple social statuses, which has been shown to have implications for mental health [40]. Therefore, research on sex and mental health should take into consideration the interaction of sex with other social statuses, including gender, socioeconomic status, and education, but until now studies have been very limited. In a very recent paper, Gibson et al. [41] explored the role of sex, gender, and education on depressive symptoms among young adults in the United States. Surprisingly, they found that femininity, and not masculinity, resulted in less depressive symptoms, both in men and women. These results stress the importance of understanding the relationship between sex, gender, and depression. These authors also found that individuals of both sexes benefit more from both masculinity and femininity with increased education, thus suggesting that those with less education may be penalized in terms of mental health by incongruence in sex-gender traits.

## 4. Neurodegenerative Diseases

### 4.1. Parkinson's Disease

Sex-related differences in Parkinson's disease (PD) have been recognized but are still poorly understood, and the impact of gender differences on the disease clinical presentation remains controversial. Both the prevalence and incidence of PD are significantly higher in men compared to women [42–44] with a male to female ratio of close to 1.6 : 1 [45, 46]. Epidemiological studies suggest that symptom onset may be later in women [44], possibly due to the neuroprotective effects of estrogen [47–52]. Sex-related differences have also been observed in the effect size of PD risk factors [53], type of motor symptoms [45, 54–56], neuropsychiatric and cognitive changes [43, 56], and development of hallucinations [57], as well as quality of life (QoL) [43, 46]. Women have shown worse capacity in activities of daily living (ADL) and more severity of levodopa-induced dyskinesia in several studies [55, 56, 58–62], while male gender is likely to predict worse rigidity score and higher risk for rapid eye movement (REM) sleep behavior disorder, dementia, and death [58, 63–65]. However, not all studies show consistent results. A recent cross-sectional survey [66] found that men reported a greater disease burden, as measured by UPDRS-III, greater daily levodopa equivalent doses, and caregiver reliance than women. The greater burden of disease score in men was significantly associated with sex even after controlling for age and disease duration. In that series, it was men that reported significantly greater difficulties with ADL, cognition, and communication, and overall PD was found to have a greater impact on the health and well-being of male patients with differences in disease experience and perception. The authors admit that the greater disease burden demonstrated by men in their survey could have been a reflection of a recruitment bias, since older patients with greater motor disability may have been overrepresented among the clinic attendees.

Two different studies found that fatigue, nervousness, sadness, constipation, restless legs, and pain were predominant in women, and daytime sleepiness, dribbling saliva, interest in sex, and sexual dysfunction in men [67, 68]. Moreover, women were more likely than men to present with tremor as initial symptom and with worse Unified Parkinson's Disease Rating Scale (UPDRS) instability score. Once more, a third study could not replicate at least some of these findings, namely, the higher prevalence of mood symptoms among women [69]. No significant gender differences were found on scores for four cardinal motor signs, neither on motor subtypes. The DEEP (Early DEtection of wEaring off in Parkinson disease) study, which assessed the frequency of wearing-off (WO) in PD patients and its impact on QoL, found that female gender, together with younger age, UPDRS part II score, and duration of anti-Parkinson treatment, was significantly associated with WO [70]. All the above-mentioned authors conclude that management of PD patients should take these gender differences into consideration, in order to achieve greater therapeutic efficacy.

### 4.2. Amyotrophic Lateral Sclerosis

McCombe and Henderson [71] have underlined some complex interactions between gender and clinical phenotype of amyotrophic lateral sclerosis (ALS), with the aim of understanding causes of gender differences that could possibly concern disease-modifying processes. Gender was reported to affect the incidence as well as the site-onset of the disease: ALS is more common in males than in females, and the proportion of patients showing limb-onset is also greater in males than in females [72]. However, the literature on this field remains scarce, especially about the role of gender in the risk of developing specific cognitive impairment in ALS. Palmieri et al. [73] tried to identify specific gender-related differences in cognitive profile in ALS through a retrospective study in a representative cohort of Italian outpatients with ALS. Independent from mood tone and clinical variables, a significantly greater executive impairment was found in female patients compared to males and control participants. Instead, no difference was observed between gender percentages in the nonexecutive cognitive dysfunction group. These results have highlighted a significant vulnerability of ALS female patients to disease-specific cognitive dysfunctions, independently of bulbar onset. As a possible explanation of the lack of correspondence of bulbar onset and executive dysfunction, Palmieri et al. propose two intriguing interpretative hypotheses that are not mutually exclusive: the role of gonadal hormones and a gender-related brain asymmetry based on preexisting morphological and functional differences between male and female brains. On the other hand, other authors had observed that bulbar onset is more common in females with ALS or that, regardless of the gender appurtenance, cognitive executive dysfunctions and bulbar onset may cooccur in patients with ALS [74].

### 4.3. Alzheimer's Disease

Women not only have a higher prevalence of Alzheimer's disease (AD) than age-matched men, but also show age-related faster decline. While it is still unknown why females have higher risk of AD than men, a review by Li and Singh [12] highlighted the important impact of sex hormones with other genetic influences on the risk of AD. Several major biological hypotheses have been formulated on sex differences in AD, such as differences in age-related sex hormone reduction (estrogen, progesterone, and testosterone), impact from risks of other diseases (diabetes, depression, and cardiovascular disease), age-related declines in brain volume, and brain glucose metabolism [75–80].

Clinical studies have shown differences in specific cognitive ability domains and risk of Alzheimer's disease (AD) between men and women at later age. However, it is important to know that sex differences in cognitive function have been observed also in adulthood and may have their basis in both organizational effects, occurring as early as during the neuronal development period and in activational effects. The rate of cognitive decline with aging is also different between the sexes [12, 75–80].

Sex differences in prevalence and severity of AD have been found. In both genders, epidemiological studies show an increased risk of AD with the age-related loss of sex steroid hormones. Clinical and preclinical studies have shown that females carry an increased risk of developing AD pathology compared to males, even after controlling for increased life span [81, 82]. Women also show significantly faster decline and greater deterioration of cognition with age than elderly male [83–85]. Postmortem human studies showed that men with AD had more pronounced pathology in right hemisphere where women with AD often had more manifest pathology [86, 87].

The hypothesis that the increase in prevalence of AD in women may be related to the more rapid decline in circulating estrogen and progesterone levels has supported the idea that interventions with estrogen may help protect against neurodegenerative diseases like AD. In fact, several observational studies have suggested since long time that estrogen may help maintain cognitive function in women with AD who are current users of estrogen/hormone therapy [88–91]. Epidemiological evidence also suggests that postmenopausal estrogen therapy reduces the risk or delays the onset of AD [92]. Notably, the Women's Health Initiative (WHI) failed to improve symptoms in women with mild to moderate AD [93]. A reanalysis of the WHI data, however, indicates that early postmenopausal treatment with estrogen can provide benefits [94, 95], as previously reported. Concerning progestins, to date, there are no clinical trials that have specifically assessed their effects when administered alone on cognitive outcomes in subjects with AD. Most studies have addressed the protective effects of progestins (typically, medroxyprogesterone acetate) when administered in conjunction with estrogen. There have been relatively few studies with small sample sizes that have addressed the effect of testosterone treatment in men with cognitive impairment and AD. One pilot study showed that testosterone treatment improved the scores at the cognitive domain of the Alzheimer's Disease Assessment Scale (ADAS Cog) and the minimental state examination (MMSE) [96], while other authors have found beneficial effects of testosterone in specific domains of cognitive function in AD patients [97]. However, not all studies show beneficial effects of testosterone. For example, Kenny et al. [98] and Lu et al. [99] failed to note any beneficial effects of testosterone. Future research should probably focus on the AD male population most likely to benefit from androgen therapy.

## 5. Multiple Sclerosis

Women are at increased risk of multiple sclerosis (MS), but men generally face a worse disease course. This apparent paradox has prompted to explore sex-related aspects of the pathophysiology of MS, as well as their possible impact on therapeutic approaches. In fact, there is increasing evidence from experimental, clinical, and epidemiological research to support a role for gender and reproductive hormones in both the onset and course of MS.

In individuals with MS, a number of genetic, environmental, and lifestyle factors have potentially sexually dimorphic effects on MS disease susceptibility and progression. These include behavioral, metabolic (obesity), genetic, and epigenetic risk factors; however, it has also been hypothesized that males have a tendency to underreport environmental and familial risk factors. Sex differences have been described in the signaling pathways that control central nervous system (CNS) autoimmunity or repair [100]. Several findings implicate a role for sex-related interactions with major histocompatibility complex (MHC) risk alleles, including epigenetic modifications [101]. Hormonal factors seem to have an impact on disease onset and course. There is evidence for hormonal modulation of MS, during transitions phases as puberty and pregnancy [102, 103]. Having the female sex and exposure to sex-specific events, such as pregnancy, are associated with an increased risk of developing clinically definite MS after a first demyelinating event. On the other hand, males are more likely to display a progressive disease onset, poor recovery after initial attacks, more rapid development of disability, and an overall more malignant course, even after controlling for sex differences in the age at onset and other confounders, while females are more likely to manifest benign MS [104]. The role of sex hormone-related factors is also supported by the potential modulatory role for hormone-based therapies, including estrogen and testosterone.

In MS patients, very strong sex effects have been described also in cognitive measures, remarkably with lesion volumes that were not different between the sexes, thus indicating that there were more severe changes in normal appearing tissue [105]. Since the white matter of male MS patients was both more extensively and more severely affected than that of female patients, the authors concluded that there may be an important and sex-specific role for white matter changes in cognitive dysfunction in MS.

In summary, sex-related differences in MS incidence and severity result from a complex interaction of hormonal, genetic, and epigenetic factors, and there is a potential for a modulatory role of hormone-based therapies, including estrogen and testosterone [106].

## 6. Epilepsy

The incidence and prevalence of unprovoked seizures are higher in men than women [107–109], and status epilepticus is more frequent in men than women [110, 111]. However, several studies have reported that generalized epilepsies are more common in women [109, 112–114], and it seems that women more frequently have idiopathic generalized epilepsy. The reasons behind this difference are not established, but sex hormones might play a role. If this assumption is true, the gender difference would be more pronounced before menopause and indeed the female preponderance was highest between 15 and 50 years and then declined with age. Overall, no gender difference was found in localization-related epilepsy, but symptomatic localization-related epilepsies were found to be more frequent in men, while cryptogenic localization-related epilepsies were found to be more frequent in women [113]. Carlson et al. [115] with the Epilepsy Phenome/Genome Project (EPGP) showed differences in several subjective ictal symptoms between males and females. The authors of these studies were not able to assess the neurobiological basis of such sex differences and could not completely rule out the possibility that the findings were biased by differences in symptom reporting and recognition; however, it is undeniable that they raise important questions about the differences that exist between sexes at the CS level.

## 7. Addictive Behaviors

Sex differences in the patterns of drug use and addition have been widely described; for instance, gender-dependent differences have been reported in the rate of initiation and frequency of misuse of addicting drugs, and the underlying mechanisms leading to these sex differences may be generalizable to other types of addictive behaviors. There is evidence of sex differences not only in abuse of addictive drugs, but also in food addiction, compulsive sexual activity, pathological gambling, Internet addiction, and physical exercise addiction [116–118]. In their recent review, Fattore et al. [118] provide an overview of potential risk factors and brain mechanisms, with a particular focus on the role of sex steroid hormone in creating sex differences in prevalence. Once again, both biological and sociocultural factors are likely to contribute to the sex differences in addictive behaviors.

### 7.1. Drug and Substances Abuse

According to the European Monitoring Centre for Drugs and Drug Addiction (EMCDDA), women are more likely than men to abuse prescribed drugs, like tranquilizers and sedatives, while men are more likely to abuse illicit drugs, such as cocaine and heroin [119]. For almost all drugs of abuse, females have an enhanced vulnerability to develop drug addiction. Prevalence of cigarette use, regular heavy episodic drinking, and marijuana use was reported to be higher for males than females overall, although gender differences vary with age [120]. It has been shown that marijuana use is strongly related to depression symptoms and cigarette use frequency in males, indicating that in males these detrimental factors converge, whereas in females they do not [121]. Higher prevalence rates of cannabis abuse/dependence and abuse/dependence criteria have been observed in males versus females, as well as in 18–24-year-old versus older cannabis users [122].

### 7.2. Behavioral Addictions

Only few studies have examined potential differences between men and women in nonsubstance behavioral addictions, although there is growing evidence indicating that eating, having sex, gambling, spending time on the Internet, or exercising can develop into compulsive behaviors. In a study using multidimensional self-report to measure addictive behaviors, men scored higher than women in exercising, gambling, and having sex, while women scored higher in compulsive shopping and food binging [123]. Concerning gambling disorders in particular, findings indicate that men are three times more likely to experience problems but also have different patterns of gambling activities. Men are more involved with Internet gambling, sports and racetrack betting, poker, and casino tables, whereas women gamble more often on scratch games. Furthermore, suicidal ideation and behaviors were reported to be more likely associated with gambling problems in women as compared to men [124]. Uncontrolled food intake tends to be more prevalent in women than in men [118], which is consistent with the slightly higher prevalence of obesity among women [WHO]. In particular, higher levels of addiction to chocolate were reported in women [125], with chocolate and sweets craving being more frequent in the perimenstrual period. Sexual addiction, also referred to as compulsive sexual behavior, has been analyzed in a very limited number of studies. A population study evaluated the occurrence of “out of control sexual experiences” in a representative sample [126], reporting that nearly 13% of men and 7% of women reported having had sexual fantasies, urges, or sexual behaviors that they considered as out of control during the past year. The DSM-5 identifies Internet Gaming Disorder as a condition warranting more clinical research and experience, before it might be considered a psychiatric disorder. The first studies on this problem showed that boys are more likely than girls to engage in playing video games or computer activities [127, 128]. More recently, in a representative sample of high school Italian students, 5% was found moderately addicted and 0.79% seriously addicted, with a significant male preponderance [129]. No gender differences were found in “addiction” to running and exercising [125, 130]. Unfortunately, to our knowledge, no study has so far investigated whether sex hormones may affect the inclination to exercising excessively.

At least for drug and alcohol addiction, sex differences in metabolism, pharmacodynamics, and pharmacokinetics have been hypothesized to play a role in the observed sex differences, which may be mediated in part by the organizational and activational effects of gonadal steroids, not surprisingly as described for affective disorders. Far less is known about the neuroendocrine factors contributing to other behavioral addictions and this is an area that deserves further investigation.

## 8. Pain

Pain is a leading public health problem in developed countries and is one of the most common reasons that individuals seek emergency care for [131]. Research regarding sex, gender, and pain has proliferated in the last decades [132], covering different topics: from preclinical studies of mechanisms contributing to sex differences in pain, human laboratory research exploring sex differences in pain perception and endogenous pain modulation, clinical and epidemiological investigations of sex differences in pain prevalence, and studies examining sex differences in responses to pain treatments. Recent publications provide thorough examinations of various areas of this literature [132–136].

In laboratory-based studies, women have been found to exhibit greater pain sensitivity, enhanced pain facilitation, and reduced pain inhibition compared with men, though the magnitude of these sex differences varies across studies [136–138]. In population-based studies, females have been consistently found to experience more severe acute and chronic pain across a range of conditions [133–135]. Large-scale epidemiological studies across multiple geographic regions find that pain is reported more frequently by women than by men [132]. Todd et al. [139] found that, for each of 10 different anatomical regions, a greater proportion of women than men reported pain in the past week, and women were significantly more likely to report chronic widespread pain. Moreover, the population prevalence of several common chronic pain conditions, including fibromyalgia, migraine, and chronic tension-type headache, is greater for women than men [136, 140]. More controversial are data about severity of pain. While some studies have reported greater pain severity among women than men [140–143], other have found no sex differences in pain severity among treatment-seeking patients [144–146]. However, it has to be considered that there might be a bias in these results as patients with less severe pain were probably underrepresented in these studies.

Some evidence suggests there are sex differences also in the responses to pharmacological and nonpharmacological pain treatments [139, 147]. In a review of 18 studies [148], a lower postoperative opioid consumption was observed among women. However, this finding is not consistent and this may depend on the type of surgical procedure [149] or on the well-known higher prevalence of side effects in women [150]. A recent meta-analysis [138] reported greater “analgesic” effects for women when restricting the analyses to patient-controlled analgesia (PCA), even greater when considering only PCA morphine studies. It is important to underline that these studies assessed opioid consumption rather than pain relief. Several investigators have also examined gender biases in the treatment of pain. Studies have demonstrated that females are considered to have greater intensity and unpleasantness of pain than males and are more likely to be given opioid treatment as reported by healthcare professionals and students [151–153]. These studies indicate gender-disparities in pain management. The literature seems to suggest that also responses to nonpharmacological treatments may differ for men and women, but the results are more variable across studies [154–158].

It has been suggested that an interaction of biological, psychological, and sociocultural factors likely contributes to sex differences in pain. Sex hormones represent a significant source of pain-related variability, impacting men and women differently. This is not surprising given the distribution of sex hormones and their receptors in areas of the peripheral and central nervous systems associated with nociceptive transmission [159, 160]. Research on sex hormones' effects on pain is still very limited, but we know that estradiol and progesterone's effects on pain sensitivity are relatively complex (both exert pronociceptive and antinociceptive effects on pain) [159, 161]. Testosterone appears to be more antinociceptive and protective [159], given the association found between decreased androgen concentrations and chronic pain [162]. Exacerbation of clinical pain has been demonstrated across the menstrual cycle for a couple of decades [163–166], with increased pain sensitivity during the luteal phase relative to the follicular phase [167]. Moreover, exogenous hormone use increases risk for some types of clinical pain [168].

Sex-related differences in pain may also reflect differences in the endogenous opioid system. For instance, there are distinct differences between men and women in pain-related activation of brain mu-opioid receptors [169], suggesting that the interactive effects of the opioidergic system with gonadal hormones may be an important determinant of sex-based differences in pain sensitivity.

Genotype may also contribute to sex differences in pain. Preclinical research consistently shows that genotype and sex interact to influence nociceptive sensitivity [170] and these findings have been extended to humans in recent years [171–173].

Various psychosocial mechanisms may play a fundamental role in sex-related differences in pain. For instance, pain coping strategies are different between men and women. Men tend to use behavioral distraction and problem-focused tactics to manage pain, while women tend to use as coping techniques social support, emotion-focused techniques, cognitive reinterpretation, and attentional focus [132, 136, 137, 174, 175]. Research has shown that catastrophizing is associated with pain and pain-related disability [176] and women engage in catastrophizing more often than men, but this may be modulated by other factors such as personality disposition [137]. Sociocultural beliefs about femininity and masculinity are also important in determining pain responses among the sexes as pain expression is generally more socially acceptable among women, and this effect may lead to biased reporting of pain.

Recently, a consensus group was created to identify priority research areas related to the influence of gender on pain assessment, treatment, and outcomes in emergency departments [177]. The three top priority areas to be investigate, as identified through the consensus process, were (I) gender differences in the pharmacological and nonpharmacological interventions for pain, including opioid tolerance, side effects, or misuse; (II) gender differences in pain severity perceptions and pain treatment preferences; (III) gender differences in pain outcomes across life span. The consensus group concluded that exploring these areas may be extremely useful for emergency physicians in order to better understand the interaction of gender and pain and appropriately address interventions in the emergency department.

## 9. Conclusions

In neurological and psychiatric disorders, understanding the biological bases of sex differences, as well as the psychosocial and cultural influences on gender differences, may be crucial for better understanding the etiology of such disorders, but more importantly, to improve therapeutic strategies. At present, the available evidence does not yet support sex-specific tailoring of treatments. However, this outcome may be conceivable in the next future. Additional research to elucidate the mechanisms driving sex differences in CNS-related diseases is needed in order to foster future interventions to reduce sex disparities in outcomes.

The aim of this review is to stimulate interest in sexual dimorphisms in the brain and brain diseases and encourage more research in the field of the implications of sex differences for treating these conditions. Recommendations shared by most of the authors we have reviewed are (I) the inclusion of both sexes in basic CNS science; (II) the exploration of sex difference as a part of the standard preclinical evaluation of therapeutics; (III) the implementation of research examining sex-specific risk factors, and (IV) the definition and use of relevant sex-specific outcome measures and therapeutic strategies.

## References

[1] Wizemann T. M., Pardu M. L. (2001). *Exploring the Biological Contributions to Human Health. Does Sex Matter?*.

[2] National Institute of Mental Health Sex differences in brain, behavior, mental health and mental disorders. http://www.nimh.nih.gov/research-priorities/scientific-meetings/2011/sex-differences-in-brain-behavior-mental-health-and-mental-disorders/index.shtml.

[3] Beery A. K., Zucker I. (2011). Sex bias in neuroscience and biomedical research. *Neuroscience and Biobehavioral Reviews*.

[4] Trabzuni D., Ramasamy A., Imran S. (2013). Widespread sex differences in gene expression and splicing in the adult human brain. *Nature Communications*.

[5] Fine C. (2010). *Delusions of Gender: The Real Science behind Sex-Differences*.

[6] Davies W. (2014). Sex differences in Attention Deficit Hyperactivity Disorder: candidate genetic and endocrine mechanisms. *Frontiers in Neuroendocrinology*.

[7] Schaafsma S. M., Pfaff D. W. (2014). Etiologies underlying sex differences in autism spectrum disorders. *Frontiers in Neuroendocrinology*.

[8] Bangasser D. A., Valentino R. J. (2014). Sex differences in stress-related psychiatric disorders: neurobiological perspectives. *Frontiers in Neuroendocrinology*.

[9] Altemus M., Sarvaiya N., Epperson C. N. (2014). Sex differences in anxiety and depression clinical perspectives. *Frontiers in Neuroendocrinology*.

[10] Ngo S. T., Steyn F. J., McCombe P. A. (2014). Gender differences in autoimmune disease. *Frontiers in Neuroendocrinology*.

[11] Gillies G. E., Pienaar I. S., Vohra S. (2014). Sex differences in Parkinson's disease. *Frontiers in Neuroendocrinology*.

[12] Li R., Singh M. (2014). Sex differences in cognitive impairment and Alzheimer's disease. *Frontiers in Neuroendocrinology*.

[13] Bartley E. J., Fillingim R. B. (2013). Sex differences in pain: a brief review of clinical and experimental findings. *British Journal of Anaesthesia*.

[14] Lai M.-C., Lombardo M. V., Ruigrok A. N. V. (2012). Cognition in males and females with autism: similarities and differences. *PLoS ONE*.

[15] Mattila M.-L., Kielinen M., Linna S.-L. (2011). Autism spectrum disorders according to DSM-IV-TR and comparison with DSM-5 draft criteria: an epidemiological study. *Journal of the American Academy of Child and Adolescent Psychiatry*.

[16] Kim Y. S., Leventhal B. L., Koh Y.-J. (2011). Prevalence of autism spectrum disorders in a total population sample. *American Journal of Psychiatry*.

[17] Rynkiewicz A., Schuller B., Marchi E. (2016). An investigation of the ‘female camouflage effect’ in autism using a computerized ADOS-2 and a test of sex/gender differences. *Molecular Autism*.

[18] Baron-Cohen S., Wheelwright S. (2004). The empathy quotient: an investigation of adults with asperger syndrome or high functioning autism, and normal sex differences. *Journal of Autism and Developmental Disorders*.

[19] Baron-Cohen S., Richler J., Bisarya D. (2003). The systemizing quotient: an investigation of adults with Asperger syndrome or high-functioning autism, and normal sex differences. *Philosophical Transactions of the Royal Society B: Biological Sciences*.

[20] Baron-Cohen S., Lombardo M. V., Auyeung B., Ashwin E., Chakrabarti B., Knickmeyer R. (2011). Why are autism spectrum conditions more prevalent in males?. *PLoS Biology*.

[21] Baron-Cohen S. (2002). The extreme male brain theory of autism. *Trends in Cognitive Sciences*.

[22] Baron-Cohen S., Cassidy S., Auyeung B. (2014). Attenuation of typical sex differences in 800 adults with autism vs. 3,900 controls. *PLoS ONE*.

[23] Frazier T. W., Georgiades S., Bishop S. L., Hardan A. Y. (2014). Behavioral and cognitive characteristics of females and males with autism in the simons simplex collection. *Journal of the American Academy of Child & Adolescent Psychiatry*.

[24] Horiuchi F., Oka Y., Uno H. (2014). Age- and sex-related emotional and behavioral problems in children with autism spectrum disorders: comparison with control children. *Psychiatry and Clinical Neurosciences*.

[25] Goddard L., Dritschel B., Howlin P. (2014). A preliminary study of gender differences in autobiographical memory in children with an autism spectrum disorder. *Journal of Autism and Developmental Disorders*.

[26] Van Wijngaarden-Cremers P. J. M., Van Eeten E., Groen W. B., Van Deurzen P. A., Oosterling I. J., Van der Gaag R. J. (2014). Gender and age differences in the core triad of impairments in autism spectrum disorders: a systematic review and meta-analysis. *Journal of Autism and Developmental Disorders*.

[27] Reinhardt V. P., Wetherby A. M., Schatschneider C., Lord C. (2015). Examination of sex differences in a large sample of young children with autism spectrum disorder and typical development. *Journal of Autism and Developmental Disorders*.

[28] Postorino V., Fatta L. M., De Peppo L. (2015). Longitudinal comparison between male and female preschool children with autism spectrum disorder. *Journal of Autism and Developmental Disorders*.

[29] Kessler R. C. (2003). Epidemiology of women and depression. *Journal of Affective Disorders*.

[30] Zender R., Olshansky E. (2009). Women's mental health: depression and anxiety. *Nursing Clinics of North America*.

[31] Horwitz A. V., White H. R. (1987). Gender role orientations and styles of pathology among adolescents. *Journal of Health and Social Behavior*.

[32] Kornstein S. G., Schatzberg A. F., Thase M. E. (2000). Gender differences in chronic major and double depression. *Journal of Affective Disorders*.

[33] Mirowsky J., Ross C. E. (2007). Life course trajectories of perceived control and their relationship to education. *American Journal of Sociology*.

[34] Noble R. E. (2005). Depression in women. *Metabolism*.

[35] Fernández-Guasti A., Fiedler J. L., Herrera L., Handa R. J. (2012). Sex, stress, and mood disorders: at the intersection of adrenal and gonadal hormones. *Hormone and Metabolic Research*.

[36] Mirowsky J., Ross C. E. (1995). Sex differences in distress: real or artifact?. *American Sociological Review*.

[37] Nolen-Hoeksema S., Larson J., Grayson C. (1999). Explaining the gender difference in depressive symptoms. *Journal of Personality and Social Psychology*.

[38] Nolen-Hoeksema S., Wisco B. E., Lyubomirsky S. (2008). Rethinking rumination. *Perspectives on Psychological Science*.

[39] Payne S. (2006). *The Health of Men and Women*.

[40] Rosenfield S. (2012). Triple jeopardy? Mental health at the intersection of gender, race, and class. *Social Science and Medicine*.

[41] Gibson P. A., Baker E. H., Milner A. N. (2016). The role of sex, gender, and education on depressive symptoms among young adults in the United States. *Journal of Affective Disorders*.

[42] Kieburtz K., Wunderle K. B. (2013). Parkinson's disease: evidence for environmental risk factors. *Movement Disorders*.

[43] Pavon J. M., Whitson H. E., Okun M. S. (2010). Parkinson's disease in women: a call for improved clinical studies and for comparative effectiveness research. *Maturitas*.

[44] Kowal S. L., Dall T. M., Chakrabarti R., Storm M. V., Jain A. (2013). The current and projected economic burden of Parkinson's disease in the United States. *Movement Disorders*.

[45] Scott B., Borgman A., Engler H., Johnels B., Aquilonius S. M. (2000). Gender differences in Parkinson's disease symptom profile. *Acta Neurologica Scandinavica*.

[46] Shulman L. M. (2007). Gender differences in Parkinson's disease. *Gender Medicine*.

[47] Ragonese P., D'Amelio M., Salemi G. (2004). Risk of Parkinson disease in women: effect of reproductive characteristics. *Neurology*.

[48] Popat R. A., Van Den Eeden S. K., Tanner C. M. (2005). Effect of reproductive factors and postmenopausal hormone use on the risk of Parkinson disease. *Neurology*.

[49] Benedetti M. D., Maraganore D. M., Bower J. H. (2001). Hysterectomy, menopause, and estrogen use preceding Parkinson's disease: an exploratory case-control study. *Movement Disorders*.

[50] Rocca W. A., Bower J. H., Maraganore D. M. (2008). Increased risk of parkinsonism in women who underwent oophorectomy before menopause. *Neurology*.

[51] Shulman L. M. (2002). Is there a connection between estrogen and Parkinson's disease?. *Parkinsonism and Related Disorders*.

[52] Saunders-Pullman R., Gordon-Elliott J., Parides M., Fahn S., Saunders H. R., Bressman S. (1999). The effect of estrogen replacement on early Parkinson's disease. *Neurology*.

[53] Palacios N., Gao X., McCullough M. L. (2012). Caffeine and risk of Parkinson's disease in a large cohort of men and women. *Movement Disorders*.

[54] Alves G., Müller B., Herlofson K. (2009). Incidence of Parkinson's disease in Norway: The Norwegian ParkWest study. *Journal of Neurology, Neurosurgery and Psychiatry*.

[55] Haaxma C. A., Bloem B. R., Borm G. F. (2007). Gender differences in Parkinson's disease. *Journal of Neurology, Neurosurgery and Psychiatry*.

[56] Lyons K. E., Hubble J. P., Tröster A. I., Pahwa R., Koller W. C. (1998). Gender differences in Parkinson's disease. *Clinical Neuropharmacology*.

[57] Zhu K., van Hilten J. J., Putter H., Marinus J. (2013). Risk factors for hallucinations in Parkinson's disease: results from a large prospective cohort study. *Movement Disorders*.

[58] Baba Y., Putzke J. D., Whaley N. R., Wszolek Z. K., Uitti R. J. (2005). Gender and the Parkinson's disease phenotype. *Journal of Neurology*.

[59] Ehrt U., Brønnick K., De Deyn P. P. (2007). Subthreshold depression in patients with Parkinson's disease and dementia—clinical and demographic correlates. *International Journal of Geriatric Psychiatry*.

[60] Accolla E., Caputo E., Cogiamanian F. (2007). Gender differences in patients with Parkinson's disease treated with subthalamic deep brain stimulation. *Movement Disorders*.

[61] Hariz G.-M., Lindberg M., Hariz M. I., Bergenheim A. T. (2003). Gender differences in disability and health-related quality of life in patients with Parkinson's disease treated with stereotactic surgery. *Acta Neurologica Scandinavica*.

[62] Rojo A., Aguilar M., Garolera M. T., Cubo E., Navas I., Quintana S. (2003). Depression in Parkinson's disease: clinical correlates and outcome. *Parkinsonism and Related Disorders*.

[63] Fernandez H. H., Lapane K. L. (2002). Predictors of mortality among nursing home residents with a diagnosis of Parkinson's disease. *Medical Science Monitor*.

[64] Galvin J. E., Pollack J., Morris J. C. (2006). Clinical phenotype of Parkinson disease dementia. *Neurology*.

[65] Scaglione C., Vignatelli L., Plazzi G. (2005). REM sleep behaviour disorder in Parkinson's disease: a questionnaire-based study. *Neurological Sciences*.

[66] Lubomski M., Rushworth R. L., Lee W., Bertram K. L., Williams D. R. (2014). Sex differences in Parkinson's disease. *Journal of Clinical Neuroscience*.

[67] Martinez-Martin P., Pecurariu C. F., Odin P. (2012). Gender-related differences in the burden of non-motor symptoms in Parkinson's disease. *Journal of Neurology*.

[68] Solla P., Cannas A., Ibba F. C. (2012). Gender differences in motor and non-motor symptoms among Sardinian patients with Parkinson's disease. *Journal of the Neurological Sciences*.

[69] Picillo M., Amboni M., Erro R. (2013). Gender differences in non-motor symptoms in early, drug-naïve Parkinson's disease. *Journal of Neurology*.

[70] Stocchi F., Antonini A., Barone P. (2014). Early Detection of wearing off in Parkinson disease: the DEEP study. *Parkinsonism and Related Disorders*.

[71] McCombe P. A., Henderson R. D. (2010). Effects of gender in amyotrophic lateral sclerosis. *Gender Medicine*.

[72] Cima V., Logroscino G., D'Ascenzo C. (2009). Epidemiology of ALS in Padova district, Italy, from 1992 to 2005. *European Journal of Neurology*.

[73] Palmieri A., Mento G., Calvo V. (2015). Female gender doubles executive dysfunction risk in ALS: a case-control study in 165 patients. *Journal of Neurology, Neurosurgery and Psychiatry*.

[74] Phukan J., Elamin M., Bede P. (2012). The syndrome of cognitive impairment in amyotrophic lateral sclerosis: a population-based study. *Journal of Neurology, Neurosurgery and Psychiatry*.

[75] Cui J., Shen Y., Li R. (2013). Estrogen synthesis and signaling pathways during aging: from periphery to brain. *Trends in Molecular Medicine*.

[76] Yue X., Lu M., Lancaster T. (2005). Brain estrogen deficiency accelerates A*β* plaque formation in an Alzheimer's disease animal model. *Proceedings of the National Academy of Sciences of the United States of America*.

[77] Cahill L. (2005). His brain, her brain. *Scientific American*.

[78] Carter C. L., Resnick E. M., Mallampalli M., Kalbarczyk A. (2012). Sex and gender differences in Alzheimer's disease: recommendations for future research. *Journal of Women's Health*.

[79] Swaab D. F., Chung W. C. J., Kruijver F. P. M., Hofman M. A., Ishunina T. A. (2001). Structural and functional sex differences in the human hypothalamus. *Hormones and Behavior*.

[80] McAllister C., Long J., Bowers A. (2010). Genetic targeting aromatase in male amyloid precursor protein transgenic mice down-regulates *β*-secretase (BACE1) and prevents Alzheimer-like pathology and cognitive impairment. *The Journal of Neuroscience*.

[81] Dye R. V., Miller K. J., Singer E. J., Levine A. J. (2012). Hormone replacement therapy and risk for neurodegenerative diseases. *International Journal of Alzheimer's Disease*.

[82] Callahan M. J., Lipinski W. J., Bian F., Durham R. A., Pack A., Walker L. C. (2001). Augmented senile plaque load in aged female *β*-amyloid precursor protein-transgenic mice. *The American Journal of Pathology*.

[83] Proust-Lima C., Amieva H., Letenneur L., Orgogozo J.-M., Jacqmin-Gadda H., Dartigues J.-F. (2008). Gender and education impact on brain aging: a general cognitive factor approach. *Psychology and Aging*.

[84] Read S., Pedersen N. L., Gatz M. (2006). Sex differences after all those years? Heritability of cognitive abilities in old age. *Journals of Gerontology Series B: Psychological Sciences and Social Sciences*.

[85] Henderson V. W., Buckwalter J. G. (1994). Cognitive deficits of men and women with Alzheimer’s disease. *Neurology*.

[86] Seshadri S., Beiser A., Kelly-Hayes M. (2006). The lifetime risk of stroke: estimates from the Framingham study. *Stroke*.

[87] Phung T. K. T., Waltoft B. L., Laursen T. M. (2010). Hysterectomy, oophorectomy and risk of dementia: a nationwide historical cohort study. *Dementia and Geriatric Cognitive Disorders*.

[88] Paganini-Hill A., Henderson V. W. (1994). Estrogen deficiency and risk of Alzheimer's disease in women. *American Journal of Epidemiology*.

[89] Henderson V. W., Paganini-Hill A., Emanuel C. K., Dunn M. E., Buckwalter J. G. (1994). Estrogen replacement therapy in older women: comparisons between Alzheimer's disease cases and nondemented control subjects. *Archives of Neurology*.

[90] Doraiswamy P. M., Bieber F., Kaiser L., Krishnan K. R., Reuning-Scherer J., Gulanski B. (1997). The Alzheimer's disease assessment scale: patterns and predictors of baseline cognitive performance in multicenter Alzheimer's disease trials. *Neurology*.

[91] Tang M.-X., Jacobs D., Stern Y. (1996). Effect of oestrogen during menopause on risk and age at onset of Alzheimer's disease. *The Lancet*.

[92] Mulnard R. A., Cotman C. W., Kawas C. (2000). Estrogen replacement therapy for treatment of mild to moderate Alzheimer disease: a randomized controlled trial. *The Journal of the American Medical Association*.

[93] Anderson G. L., Limacher M., Assaf A. R. (2004). Effects of conjugated equine estrogen in postmenopausal women with hysterectomy: the Women’s Health Initiative randomized controlled trial. *Journal of the American Medical Association*.

[94] Hsia J., Langer R. D., Manson J. E. (2006). Conjugated equine estrogens and coronary heart disease: the Womens Health initiative. *Archives of Internal Medicine*.

[95] North American Menopause Society (2012). The 2012 hormone therapy position statement of the North American Menopause Society. *Menopause*.

[96] Tan R. S., Pu S. J. (2003). A pilot study on the effects of testosterone in hypogonadal aging male patients with Alzheimer's disease. *Aging Male*.

[97] Cherrier M. M., Matsumoto A. M., Amory J. K. (2005). Testosterone improves spatial memory in men with Alzheimer disease and mild cognitive impairment. *Neurology*.

[98] Kenny A. M., Fabregas G., Song C., Biskup B., Bellantonio S. (2004). Effects of testosterone on behavior, depression, and cognitive function in older men with mild cognitive loss. *The Journals of Gerontology—Series A: Biological Sciences and Medical Sciences*.

[99] Lu P. H., Masterman D. A., Mulnard R. (2006). Effects of testosterone on cognition and mood in male patients with mild Alzheimer disease and healthy elderly men. *Archives of Neurology*.

[100] Krementsov D. N., Noubade R., Dragon J. A., Otsu K., Rincon M., Teuscher C. (2014). Sex-specific control of central nervous system autoimmunity by p38 mitogen-activated protein kinase signaling in myeloid cells. *Annals of Neurology*.

[101] Sadovnick A. D. (2013). Differential effects of genetic susceptibility factors in males and females with multiple sclerosis. *Clinical Immunology*.

[102] Chitnis T. (2013). Role of puberty in multiple sclerosis risk and course. *Clinical Immunology*.

[103] Confavreux C., Hutchinson M., Hours M. M., Cortinovis-Tourniaire P., Moreau T. (1998). Rate of pregnancy-related relapse in multiple sclerosis. Pregnancy in Multiple Sclerosis Group. *The New England Journal of Medicine*.

[104] Bove R., Chitnis T. (2013). Sexual disparities in the incidence and course of MS. *Clinical Immunology*.

[105] Schoonheim M. M., Vigeveno R. M., Rueda Lopes F. C. (2014). Sex-specific extent and severity of white matter damage in multiple sclerosis: implications for cognitive decline. *Human Brain Mapping*.

[106] Bove R., Chitnis T. (2014). The role of gender and sex hormones in determining the onset and outcome of multiple sclerosis. *Multiple Sclerosis Journal*.

[107] Hauser W. A., Engel J., Pedley T. A. (1997). Incidence and prevalence. *Epilepsy: A Comprehensive Textbook*.

[108] Hauser W. A., Annegers J. F., Kurland L. T. (1993). Incidence of epilepsy and unprovoked seizures in Rochester, Minnesota: 1935–1984. *Epilepsia*.

[109] Kotsopoulos I. A. W., van Merode T., Kessels F. G. H., De Krom M. C. T. F. M., Knottnerus J. A. (2002). Systematic review and meta-analysis of incidence studies of epilepsy and unprovoked seizures. *Epilepsia*.

[110] Coeytaux A., Jallon P., Galobardes B., Morabia A. (2000). Incidence of status epilepticus in French-speaking Switzerland: (EPISTAR). *Neurology*.

[111] Hesdorffer D. C., Logroscino G., Cascino G., Annegers J. F., Hauser W. A. (1998). Incidence of status epilepticus in Rochester, Minnesota, 1965–1984. *Neurology*.

[112] Diagan M., Bhalla D., Ngoungou E., Preux P.-M., Benbadis S. R., Beran R. G., Berg A. T. (2010). Epidemiology of epilepsies in resource-poor countries. *Atlas of Epilepsies*.

[113] Christensen J., Kjeldsen M. J., Andersen H., Friis M. L., Sidenius P. (2005). Gender differences in epilepsy. *Epilepsia*.

[114] Waaler P. E., Blom B. H., Skeidsvoll H., Mykletun A. (2000). Prevalence, classification, and severity of epilepsy in children in Western Norway. *Epilepsia*.

[115] Carlson C., Dugan P., Kirsch H. E., Friedman D. (2014). Sex differences in seizure types and symptoms. *Epilepsy and Behavior*.

[116] Brydges N. M., Holmes M. C., Harris A. P., Cardinal R. N., Hall J. (2015). Early life stress produces compulsive-like, but not impulsive, behavior in females. *Behavioral Neuroscience*.

[117] Meulemans S., Pribis P., Grajales T., Krivak G. (2014). Gender differences in exercise dependence and eating disorders in young adults: a path analysis of a conceptual model. *Nutrients*.

[118] Fattore L., Melis M., Fadda P., Fratta W. (2014). Sex differences in addictive disorders. *Frontiers in Neuroendocrinology*.

[119] European Monitoring Centre for Drugs and Drug Addiction (EMCDDA)

[120] Evans-Polce R. J., Vasilenko S. A., Lanza S. T. (2015). Changes in gender and racial/ethnic disparities in rates of cigarette use, regular heavy episodic drinking, and marijuana use: ages 14 to 32. *Addictive Behaviors*.

[121] Crane N. A., Langenecker S. A., Mermelstein R. J. (2015). Gender differences in the associations among marijuana use, cigarette use, and symptoms of depression during adolescence and young adulthood. *Addictive Behaviors*.

[122] Delforterie M. J., Creemers H. E., Agrawal A., Lynskey M. T., Jak S., Huizink A. C. (2015). The influence of age and gender on the likelihood of endorsing cannabis abuse/dependence criteria. *Addictive Behaviors*.

[123] MacLaren V. V., Best L. A. (2010). Multiple addictive behaviors in young adults: student norms for the Shorter PROMIS questionnaire. *Addictive Behaviors*.

[124] Husky M. M., Michel G., Richard J.-B., Guignard R., Beck F. (2015). Gender differences in the associations of gambling activities and suicidal behaviors with problem gambling in a nationally representative French sample. *Addictive Behaviors*.

[125] Greenberg J. L., Lewis S. E., Dodd D. K. (1999). Overlapping addictions and self-esteem among college men and women. *Addictive Behaviors*.

[126] Skegg K., Nada-Raja S., Dickson N., Paul C. (2010). Perceived ‘out of control’ sexual behavior in a cohort of young adults from the Dunedin Multidisciplinary Health and Development study. *Archives of Sexual Behavior*.

[127] Johansson A., Götestam K. G. (2004). Internet addiction: characteristics of a questionnaire and prevalence in Norwegian youth (12–18 years). *Scandinavian Journal of Psychology*.

[128] Ko C.-H., Liu G.-C., Yen J.-Y., Yen C.-F., Chen C.-S., Lin W.-C. (2013). The brain activations for both cue-induced gaming urge and smoking craving among subjects comorbid with Internet gaming addiction and nicotine dependence. *Journal of Psychiatric Research*.

[129] Poli R., Agrimi E. (2012). Internet addiction disorder: prevalence in an Italian student population. *Nordic Journal of Psychiatry*.

[130] Furst D. M., Germone K. (1993). Negative addiction in male and female runners and exercisers. *Perceptual and Motor Skills*.

[131] Cordell W. H., Keene K. K., Giles B. K., Jones J. B., Jones J. H., Brizendine E. J. (2002). The high prevalence of pain in emergency medical care. *The American Journal of Emergency Medicine*.

[132] Fillingim R. B., King C. D., Ribeiro-Dasilva M. C., Rahim-Williams B., Riley J. L. (2009). Sex, gender, and pain: a review of recent clinical and experimental findings. *Journal of Pain*.

[133] Bernardes S. F., Keogh E., Lima M. L. (2008). Bridging the gap between pain and gender research: a selective literature review. *European Journal of Pain*.

[134] Hurley R. W., Adams M. C. B. (2008). Sex, gender, and pain: an overview of a complex field. *Anesthesia and Analgesia*.

[135] Mogil J. S. (2012). Sex differences in pain and pain inhibition: multiple explanations of a controversial phenomenon. *Nature Reviews Neuroscience*.

[136] Racine M., Tousignant-Laflamme Y., Kloda L. A., Dion D., Dupuis G., Choinire M. (2012). A systematic literature review of 10 years of research on sex/gender and experimental pain perception—part 1: are there really differences between women and men?. *Pain*.

[137] Racine M., Tousignant-Laflamme Y., Kloda L. A., Dion D., Dupuis G., Choinire M. (2012). A systematic literature review of 10 years of research on sex/gender and pain perception—part 2: do biopsychosocial factors alter pain sensitivity differently in women and men?. *Pain*.

[138] Niesters M., Dahan A., Kest B. (2010). Do sex differences exist in opioid analgesia? A systematic review and meta-analysis of human experimental and clinical studies. *Pain*.

[139] Todd K. H., Funk K. G., Funk J. P., Bonacci R. (1996). Clinical significance of reported changes in pain severity. *Annals of Emergency Medicine*.

[140] Fillingim R. B., Doleys D. M., Edwards R. R., Lowery D. (2003). Clinical characteristics of chronic back pain as a function of gender and oral opioid use. *Spine*.

[141] Keefe F. J., Lefebvre J. C., Egert J. R., Affleck G., Sullivan M. J., Caldwell D. S. (2000). The relationship of gender to pain, pain behavior, and disability in osteoarthritis patients: The role of catastrophizing. *Pain*.

[142] Barnabe C., Bessette L., Flanagan C. (2012). Sex differences in pain scores and localization in inflammatory arthritis: a systematic review and metaanalysis. *The Journal of Rheumatology*.

[143] Tang Y.-R., Yang W.-W., Wang Y.-L., Lin L. (2012). Sex differences in the symptoms and psychological factors that influence quality of life in patients with irritable bowel syndrome. *European Journal of Gastroenterology and Hepatology*.

[144] Edwards R. R., Augustson E., Fillingim R. (2003). Differential relationships between anxiety and treatment-associated pain reduction among male and female chronic pain patients. *Clinical Journal of Pain*.

[145] Robinson M. E., Wise E. A., Riley J. L., Atchison J. W. (1998). Sex differences in clinical pain: a multisample study. *Journal of Clinical Psychology in Medical Settings*.

[146] Turk D. C., Okifuji A. (1999). Does sex make a difference in the prescription of treatments and the adaptation to chronic pain by cancer and non-cancer patients?. *Pain*.

[147] Probst B. D., Lyons E., Leonard D., Esposito T. J. (2005). Factors affecting emergency department assessment and management of pain in children. *Pediatric Emergency Care*.

[148] Miaskowski C., Gear R. W., Levine J. D., Fillingim R. B. (2000). Sex-related differences in analgesic responses. *Sex, Gender, and Pain*.

[149] Chang K.-Y., Tsou M.-Y., Chan K.-H., Sung C.-S., Chang W.-K. (2006). Factors affecting patient-controlled analgesia requirements. *Journal of the Formosan Medical Association*.

[150] Fillingim R. B., Ness T. J., Glover T. L. (2005). Morphine responses and experimental pain: sex differences in side effects and cardiovascular responses but not analgesia. *The Journal of Pain*.

[151] Alqudah A. F., Hirsh A. T., Stutts L. A., Scipio C. D., Robinson M. E. (2010). Sex and race differences in rating others' pain, pain-related negative mood, pain coping, and recommending medical help. *Journal of Cyber Therapy and Rehabilitation*.

[152] Wandner L. D., Stutts L. A., Alqudah A. F. (2010). Virtual human technology: patient demographics and healthcare training factors in pain observation and treatment recommendations. *Journal of Pain Research*.

[153] Hirsh A. T., George S. Z., Robinson M. E. (2009). Pain assessment and treatment disparities: a virtual human technology investigation. *Pain*.

[154] Keogh E., Herdenfeldt M. (2002). Gender, coping and the perception of pain. *Pain*.

[155] Keogh E., Bond F. W., Hanmer R., Tilston J. (2005). Comparing acceptance- and control-based coping instructions on the cold-pressor pain experiences of healthy men and women. *European Journal of Pain*.

[156] Sternberg W. F., Bokat C., Kass L., Alboyadjian A., Gracely R. H. (2001). Sex-dependent components of the analgesia produced by athletic competition. *Journal of Pain*.

[157] Keogh E., McCracken L. M., Eccleston C. (2005). Do men and women differ in their response to interdisciplinary chronic pain management?. *Pain*.

[158] Pieh C., Altmeppen J., Neumeier S., Loew T., Angerer M., Lahmann C. (2012). Gender differences in outcomes of a multimodal pain management program. *Pain*.

[159] Craft R. M. (2007). Modulation of pain by estrogens. *Pain*.

[160] Craft R. M., Mogil J. S., Aloisi A. M. (2004). Sex differences in pain and analgesia: the role of gonadal hormones. *European Journal of Pain*.

[161] Smith Y. R., Stohler C. S., Nichols T. E., Bueller J. A., Koeppe R. A., Zubieta J.-K. (2006). Pronociceptive and antinociceptive effects of estradiol through endogenous opioid neurotransmission in women. *The Journal of Neuroscience*.

[162] Cairns B. E., Gazerani P. (2009). Sex-related differences in pain. *Maturitas*.

[163] LeResche L., Mancl L., Sherman J. J., Gandara B., Dworkin S. F. (2003). Changes in temporomandibular pain and other symptoms across the menstrual cycle. *Pain*.

[164] Ader D. N., South-Paul J., Adera T., Deuster P. A. (2001). Cyclical mastalgia: prevalence and associated health and behavioral factors. *Journal of Psychosomatic Obstetrics and Gynecology*.

[165] Silberstein S. D., Merriam G. R. (1993). Sex hormones and headache. *Journal of Pain and Symptom Management*.

[166] Huerta-Franco M. R., Malacara J. M. (1993). Association of physical and emotional symptoms with the menstrual cycle and life-style. *Journal of Reproductive Medicine*.

[167] Riley J. L., Robinson M. E., Wise E. A., Price D. (1999). A meta-analytic review of pain perception across the menstrual cycle. *Pain*.

[168] LeResche L., Saunders K., Von Korff M. R., Barlow W., Dworkin S. F. (1997). Use of exogenous hormones and risk of temporo-mandibular disorder pain. *Pain*.

[169] Zubieta J.-K., Smith Y. R., Bueller J. A. (2002). *μ*-Opioid receptor-mediated antinociceptive responses differ in men and women. *The Journal of Neuroscience*.

[170] Mogil J. S., Fillingim R. B. (2000). Interactions between sex and genotype in the mediation and modulation of nociception in rodents. *Sex, Gender, and Pain (Progress in Pain Research and management)*.

[171] Mogil J. S., Wilson S. G., Chesler E. J. (2003). The melanocortin-1 receptor gene mediates female-specific mechanisms of analgesia in mice and humans. *Proceedings of the National Academy of Sciences of the United States of America*.

[172] Fillingim R. B., Kaplan L., Staud R. (2005). The A118G single nucleotide polymorphism of the *μ*-opioid receptor gene (OPRM1) is associated with pressure pain sensitivity in humans. *Journal of Pain*.

[173] Olsen M. B., Jacobsen L. M., Schistad E. I. (2012). Pain intensity the first year after lumbar disc herniation is associated with the A118G polymorphism in the opioid receptor mu 1 gene: evidence of a sex and genotype interaction. *Journal of Neuroscience*.

[174] Unruh A. M., Judith Ritchie R. N., Merskey H. (1999). Does gender affect appraisal of pain and pain coping strategies?. *Clinical Journal of Pain*.

[175] Keogh E., Eccleston C. (2006). Sex differences in adolescent chronic pain and pain-related coping. *Pain*.

[176] Keefe F. J., Brown G. K., Wallston K. A., Caldwell D. S. (1989). Coping with rheumatoid arthritis pain: catastrophizing as a maladaptive strategy. *Pain*.

[177] Patel R., Biros M. H., Moore J., Miner J. R. (2014). Gender differences in patient-described pain, stress, and anxiety among patients undergoing treatment for painful conditions in the emergency department. *Academic Emergency Medicine*.

